# Perioperative risk stratification after resection of brain metastases: internal development and validation of the dominant lesion surgery score in a 20-year single-center cohort

**DOI:** 10.1007/s11060-026-05531-5

**Published:** 2026-03-23

**Authors:** Hasan Ali Aydın, Emrah Keskin, Murat Kalaycı

**Affiliations:** Department of Neurosurgery, School of Medicine, Zonguldak Bülent Ecevit University, Zonguldak, 67600 Türkiye

**Keywords:** Brain metastases, Surgical resection, Prognostic modeling, Risk stratification, Decision-curve analysis

## Abstract

**Objective:**

To examine whether intracranial lesion count remains associated with survival after resection of brain metastases and to develop an internally validated perioperative risk-stratification model for early mortality in a surgically treated cohort.

**Methods:**

We conducted a retrospective single-center cohort study of adults who underwent surgical resection for histologically confirmed brain metastases between 2002 and 2024. Overall survival was analyzed using Kaplan–Meier methods and multivariable Cox regression. The Dominant Lesion Surgery Score (DLSS) was derived from variables available during the perioperative period that were associated with early mortality and was evaluated for 6- and 12-month mortality using receiver operating characteristic analysis. Internal validation was performed with bootstrap resampling, and model performance was further assessed using calibration and decision-curve analysis.

**Results:**

Among 189 surgically treated patients, lesion count was not the strongest variable associated with survival after multivariable adjustment, whereas extent of resection and histology-defined tumor subtype showed stronger associations within this selected cohort. DLSS demonstrated moderate discrimination for 6- and 12-month mortality, with stable optimism-corrected performance after bootstrap validation. Calibration analysis showed acceptable agreement between predicted and observed mortality. Decision-curve analysis suggested potential net benefit across clinically relevant threshold probabilities. A DLSS cutoff of ≥ 2 identified a subgroup with higher 12-month mortality.

**Conclusion:**

Among surgically treated patients with brain metastases, lesion count alone may be insufficient to characterize postoperative risk. DLSS should be regarded as an exploratory internally derived model for perioperative risk stratification rather than a standalone tool for treatment selection, and external validation is required before broader clinical use.

## Introduction

Brain metastases represent the most common intracranial tumors in adults and remain a major source of neurological morbidity and functional deterioration among patients with systemic malignancies. Over the past decade, the therapeutic landscape of brain metastasis management has evolved substantially with the widespread adoption of stereotactic radiosurgery (SRS), refinement of postoperative cavity-directed irradiation strategies, and the increasing availability of systemic therapies with clinically meaningful central nervous system (CNS) activity in selected tumor subtypes. Contemporary multidisciplinary guidelines therefore emphasize individualized treatment strategies that integrate neurological status, systemic disease burden, and expected local control rather than relying exclusively on historical treatment thresholds [1–3].

Historically, intracranial lesion count has served as a convenient surrogate for treatment selection and has frequently functioned as an informal threshold influencing surgical candidacy. However, lesion number alone provides an incomplete representation of metastatic disease biology and clinical urgency. It does not account for the presence of dominant symptomatic lesions, acute neurological deterioration caused by mass effect, diagnostic uncertainty requiring tissue confirmation, or the strategic cytoreductive role that surgery may play within a broader multimodal treatment plan. Current consensus recommendations acknowledge that surgical resection may be appropriate not only for solitary metastases but also for selected patients harboring dominant symptomatic lesions or life-threatening mass effect, even when additional intracranial metastases are present, provided that surgery offers rapid decompression, diagnostic clarification, or meaningful local cytoreduction within a coordinated oncologic strategy [1–3]. In parallel, advances in SRS and postoperative radiation delivery have further reduced the historical reliance on rigid lesion-count thresholds by enabling effective local treatment of both intact and resected metastases [2, 4].

The potential role of surgery in carefully selected patients has been supported by a longstanding evidence base. Randomized studies performed prior to the widespread adoption of SRS demonstrated improved outcomes with surgical resection followed by radiotherapy compared with radiotherapy alone in patients with a single brain metastasis [5, 6]. However, the central clinical question in contemporary practice has shifted. Rather than asking whether surgery benefits patients with a solitary lesion, current decision-making increasingly focuses on identifying which patients derive meaningful benefit from resection of a dominant lesion within a multidisciplinary treatment paradigm that incorporates radiation and systemic therapies.

Despite major advances in systemic treatment and radiation technology, many retrospective surgical series continue to report aggregate survival outcomes without clearly operationalizing the concept of appropriate surgical selection. Prognostic frameworks such as diagnosis-specific graded prognostic assessments provide estimates of overall survival based largely on demographic and oncologic variables but often lack direct applicability to perioperative neurosurgical decision-making. In particular, clinically interpretable models specifically addressing perioperative risk stratification after dominant-lesion resection remain limited in the literature.

In this context, we conducted a 20-year retrospective analysis of surgically treated brain metastases at a tertiary neurosurgical center. The study was designed to examine outcomes within a surgically selected cohort treated in a real-world multidisciplinary environment rather than to define universal treatment thresholds. The objectives were threefold: first, to evaluate whether intracranial lesion number remains associated with survival after surgical treatment in contemporary multimodal practice; second, to explore the relative contribution of operative completeness and tumor biology to outcomes in this setting; and third, to develop and internally validate a pragmatic Dominant Lesion Surgery Score (DLSS) for perioperative risk stratification of early postoperative mortality. By combining long-term survival analysis with discrimination, calibration, and decision-curve assessment, the present study seeks to provide a quantitative framework for exploring perioperative risk patterns following dominant-lesion resection while acknowledging the limitations inherent to retrospective single-center surgical datasets.

## Materials and methods

### Study design and cohort definition

This retrospective cohort study included consecutive adult patients who underwent surgical resection for histologically confirmed brain metastases at a single tertiary neurosurgical center between January 2002 and December 2024. Patients were identified through institutional neurosurgical operative databases and oncology registries.

Eligibility criteria required: (1) histopathological confirmation of metastatic brain disease following surgical resection, and (2) availability of complete perioperative clinical, radiological, treatment, and survival information.

Patients who underwent biopsy without resection, those managed exclusively with non-surgical local therapies (e.g., radiosurgery alone), or those lacking reliable survival follow-up were excluded.

The study cohort therefore represents a surgically selected real-world population treated within a multidisciplinary neuro-oncology framework rather than the broader population of patients with brain metastases.

### Data acquisition and variable definitions

Clinical, radiological, and treatment variables were extracted from electronic medical records, operative reports, and multidisciplinary tumor board documentation.

Collected variables included age, sex, primary tumor subtype, intracranial lesion number and distribution, lesion location, surgical indication, extent of resection, adjuvant treatments, treatment era, recurrence status, and survival outcomes.

Primary tumor origin was categorized as lung, breast, gastrointestinal, genitourinary, or other solid tumors. For analytic purposes, tumor subtypes were also grouped according to histology-defined biological aggressiveness, based on previously reported survival patterns and responsiveness to systemic therapy.

Intracranial disease burden was categorized as solitary versus multiple metastases based on preoperative neuroimaging.

Extent of resection was classified as:

- **Gross total resection (GTR)** – no visible residual tumor on early postoperative imaging and operative documentation.
- **Subtotal resection (STR)** – presence of residual disease on postoperative imaging or documented intraoperative subtotal removal.

### Surgical strategy and temporal stratification

Surgical intent was determined from operative reports and tumor board documentation and categorized as:

- decompressive.
- cytoreductive.
- diagnostic.
- combined indications.

Given substantial evolution in systemic therapies and radiation techniques during the study period, patients were stratified into two predefined treatment eras:

Early era: 2002–2011.

Contemporary era: 2012–2024.

Postoperative adjuvant treatments—including stereotactic radiosurgery (SRS), whole-brain radiotherapy (WBRT), and systemic therapies—were recorded. Because these treatments occur after surgery and may be influenced by postoperative recovery or early mortality, they were interpreted cautiously in prognostic analyses.

### Outcomes

The primary endpoint was overall survival (OS), defined as the interval between the date of surgical resection and death from any cause or last clinical follow-up. Secondary endpoints included local recurrence and perioperative complications. For prognostic modeling, early mortality at 6 and 12 months after surgery was predefined as clinically relevant binary outcomes. To reduce bias in time-dependent analyses, patients censored before the predefined mortality time points were excluded from the corresponding discrimination analyses.

### Development of the Dominant Lesion Surgery Score (DLSS)

The Dominant Lesion Surgery Score (DLSS) was developed as an exploratory perioperative risk-stratification model aimed at identifying patients at increased risk of early mortality following surgical treatment.

Candidate predictors were selected a priori based on clinical plausibility and observed associations with survival in preliminary multivariable modeling.

The candidate variables included:

- extent of resection (STR vs. GTR).
- presence of multiple intracranial metastases.
- histology-defined aggressive primary tumor subtype.
- absence of adjuvant therapy.

Independent predictors identified in multivariable Cox regression were incorporated into a simplified point-based scoring system. Point assignments reflected the relative magnitude of association with early mortality while maintaining clinical interpretability.

The final DLSS ranged from 0 to 7 points, with higher scores indicating greater predicted risk of early postoperative mortality.

Because some candidate variables may be influenced by postoperative clinical course, DLSS was interpreted as a perioperative risk-stratification model rather than a preoperative treatment-selection instrument.

## Model performance and internal validation

### Discrimination

The discriminative performance of DLSS for predicting 6- and 12-month mortality was evaluated using receiver operating characteristic (ROC) curve analysis and area under the curve (AUC) estimates. Optimal risk thresholds were identified using the Youden index.

### Internal validation

Model stability and potential overfitting were assessed using bootstrap resampling with ≥ 500 iterations. Optimism-corrected AUC estimates were calculated to provide internally validated performance metrics.

### Calibration

Calibration was assessed by comparing predicted versus observed mortality probabilities across deciles of predicted risk. Calibration curves were constructed to evaluate agreement between estimated and observed event rates.

### Decision-curve analysis

Clinical relevance of DLSS predictions was explored using decision-curve analysis (DCA). Net benefit was estimated across clinically relevant threshold probabilities and compared with treat-all and treat-none strategies.

### Survival modeling

Overall survival was estimated using the Kaplan–Meier method and compared using the log-rank test.

Potential prognostic variables were evaluated using univariate and multivariable Cox proportional hazards regression models, with hazard ratios (HRs) and 95% confidence intervals (CIs) reported.

The proportional hazards assumption was verified prior to final model construction.

### Statistical considerations

Continuous variables are presented as means or medians as appropriate, while categorical variables are expressed as frequencies and percentages.

All statistical tests were two-sided, and *p* < 0.05 was considered statistically significant.

Statistical analyses were performed using SPSS software, and validation procedures were implemented using resampling techniques.

### Ethical approval

The study was approved by the Institutional Ethics Committee (Approval No: 2025/10; May 21, 2025) and conducted in accordance with the Declaration of Helsinki.

Given the retrospective design and anonymized data analysis, the requirement for individual informed consent was waived by the ethics committee.

## Results

### Cohort characteristics

A total of 189 consecutive patients who underwent surgical resection for histologically confirmed brain metastases were included in the final analysis. Baseline demographic and clinical characteristics are summarized in Table 1.

The median age at surgery was 62 years, and 78.3% of patients were male. Lung carcinoma represented the predominant primary tumor (61.9%), followed by breast (6.9%), gastrointestinal (4.8%), genitourinary (4.2%), and other solid tumors (21.7%).

Most patients presented with a solitary intracranial metastasis (73.0%), whereas 27.0% harbored multiple lesions on preoperative imaging. Gross total resection (GTR) was achieved in 53.4%, while 46.6% underwent subtotal resection (STR). The majority of operations occurred during the contemporary treatment era (2012–2024; 78.8%). The median overall survival for the entire surgically treated cohort was 6.0 months.

**Table 1:** Baseline clinical and surgical characteristics (*n* = 189)

Variable	Value
Median age (years)	62
Male sex	148 (78.3%)
Solitary metastasis	138 (73.0%)
Multiple metastases	51 (27.0%)
Lung primary	117 (61.9%)
Breast primary	13 (6.9%)
Gastrointestinal primary	9 (4.8%)
Genitourinary primary	8 (4.2%)
Other solid tumors	41 (21.7%)
Gross total resection	101 (53.4%)
Subtotal resection	88 (46.6%)
Early era (2002–2011)	40 (21.2%)
Contemporary era (2012–2024)	149 (78.8%)
Median overall survival	6.0 months

### Survival according to lesion burden and surgical extent

**Fig. 1:**
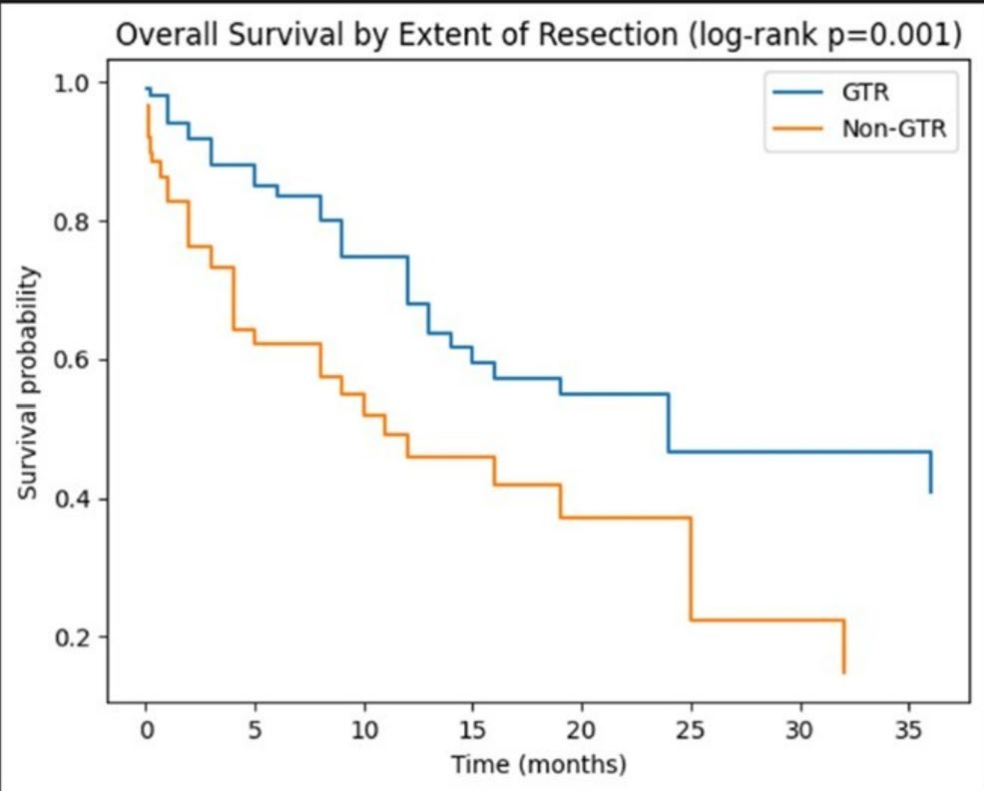
Overall survival according to extent of resection

Kaplan–Meier analysis demonstrated significantly longer survival among patients who underwent gross total resection (GTR) compared with those with non-GTR resections (Fig. 1).

Median survival was 24 months in the GTR group and 11 months in the non-GTR group (log-rank *p* = 0.001). The difference reflects substantial survival heterogeneity and right-censoring within the cohort.

Patients presenting with solitary metastases demonstrated longer unadjusted survival compared with those harboring multiple lesions. However, the magnitude of this difference was reduced after adjustment for other clinical variables.

### Multivariable survival analysis

Multivariable Cox proportional hazards regression was performed to evaluate independent associations with overall survival (Table 2).

Within the multivariable model, extent of resection emerged as the strongest variable associated with survival, with gross total resection associated with a lower hazard of death (HR 0.41; 95% CI 0.24–0.69; *p* = 0.001).

Multiple metastases demonstrated a statistically significant but modest association with survival (HR 0.54; 95% CI 0.30–0.99; *p* = 0.046). The magnitude and direction of this association should be interpreted cautiously given the surgically selected nature of the cohort and the potential influence of treatment selection patterns. Treatment era and most primary tumor subtypes were not independently associated with survival.

**Table 2:** Multivariable Cox proportional hazards analysis for overall survival

Variable	Hazard Ratio	95% CI	*p*-value
Multiple metastases (vs. solitary)	**0.54**	**0.30–0.99**	**0.046**
Gross total resection (vs. non-GTR)	**0.41**	**0.24–0.69**	**0.001**
Contemporary era (vs. early)	1.18	0.60–2.29	0.624
Breast vs. lung	1.31	0.50–3.40	0.582
Gastrointestinal vs. lung	1.42	0.53–3.78	0.484
Genitourinary vs. lung	1.95	0.74–5.10	0.177
Other vs. lung	1.63	0.94–2.82	0.083

### Development of the dominant lesion surgery score (DLSS)

The Dominant Lesion Surgery Score (DLSS) was constructed using four perioperative predictors identified from multivariable modeling:

- Subtotal resection.
- Presence of multiple metastases.
- Histology-defined aggressive primary tumor subtype.
- Absence of adjuvant therapy.

The score ranged from 0 to 7 points, with higher scores reflecting greater predicted risk of early mortality.

### Discriminatory performance

**Fig. 2:**
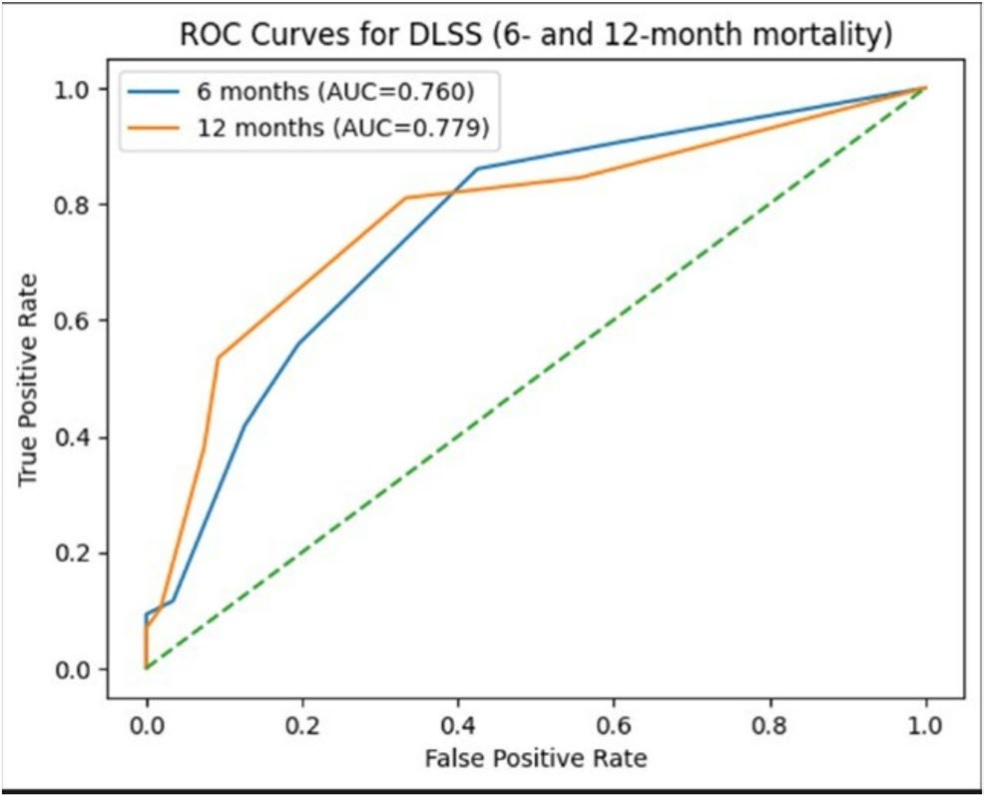
Receiver operating characteristic (ROC) curves of DLSS for 6- and 12-month mortality

DLSS demonstrated moderate discriminatory ability comparable to other clinical prognostic models in brain metastases literature for early mortality prediction (Fig. 2):

- 6-month mortality: AUC = 0.760.
- 12-month mortality: AUC = 0.779.

Using the Youden index, the optimal cutoff was identified as DLSS ≥ 2, which defined a higher-risk subgroup for early mortality.

### Internal validation

Internal validation using bootstrap resampling (1,000 iterations) demonstrated stable optimism-corrected AUC estimates for both 6- and 12-month mortality, suggesting limited overfitting within the present dataset.

### Calibration

**Fig. 3:**
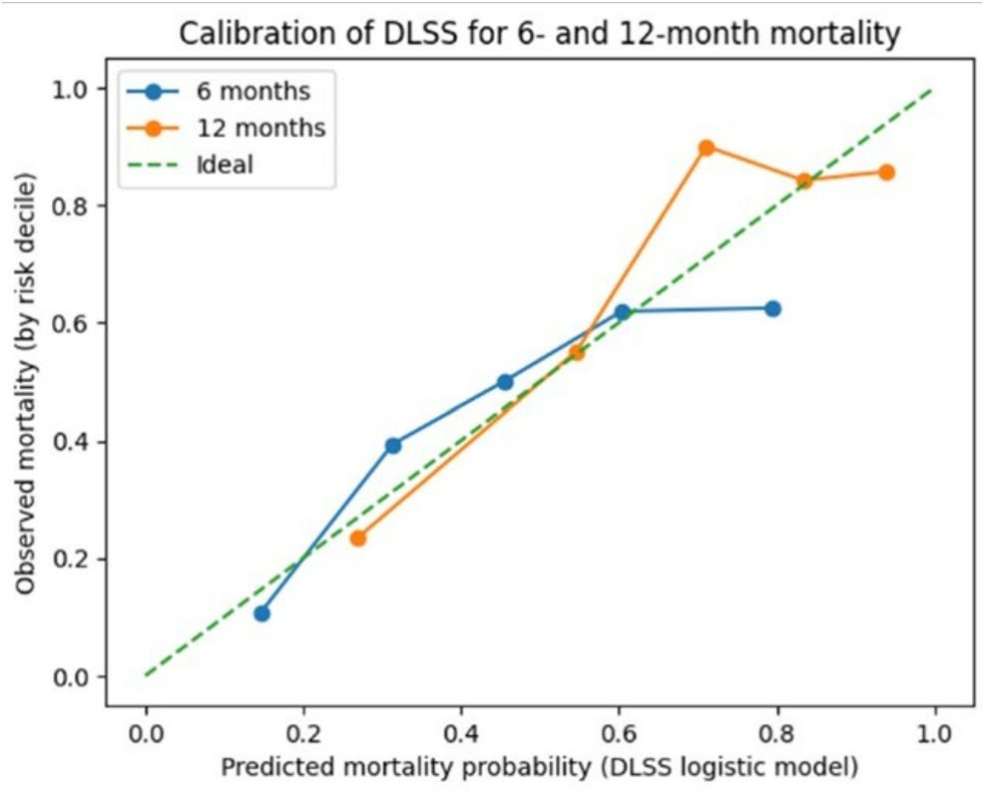
Calibration of the dominant lesion surgery score for 6- and 12-month mortality

Calibration analysis demonstrated acceptable agreement between predicted and observed mortality probabilities across deciles of predicted risk for both time points (Fig. 3). Calibration curves showed no major systematic deviation between estimated and observed event rates.

### Decision-curve analysis

**Fig. 4:**
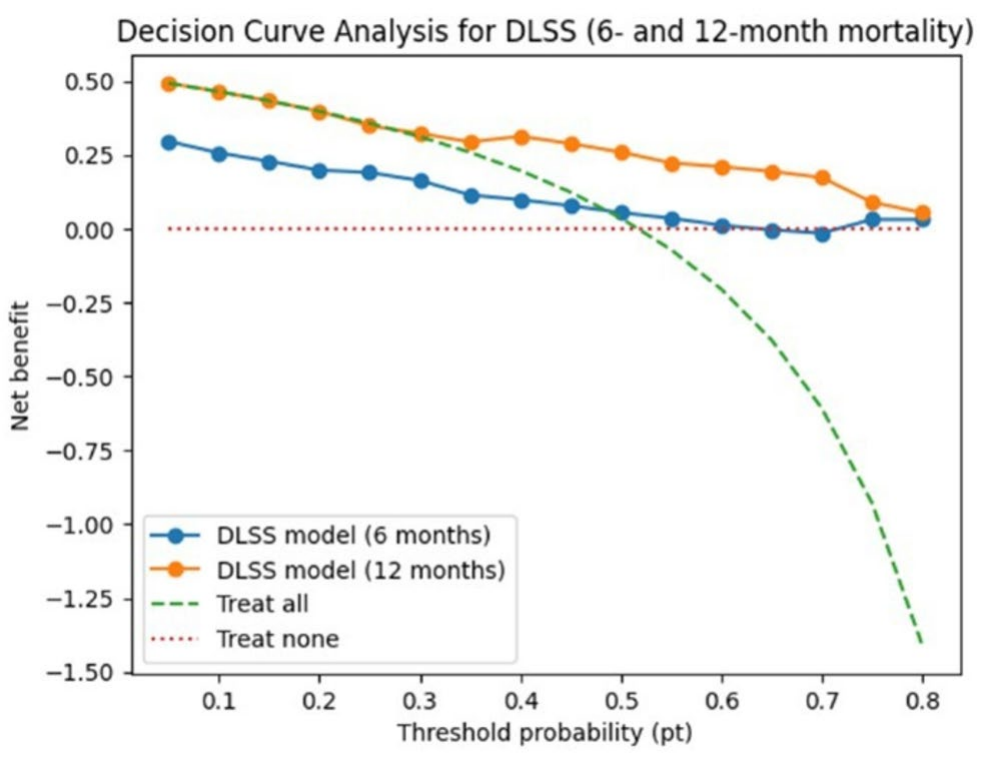
Decision-curve analysis of DLSS for early mortality prediction

Decision-curve analysis indicated potential net benefit for DLSS-based risk stratification across threshold probabilities between approximately 10% and 50% for early mortality (Fig. 4). Within this range, the model showed higher net benefit than both treat-all and treat-none strategies.

### DLSS-based risk stratification

**Fig. 5:**
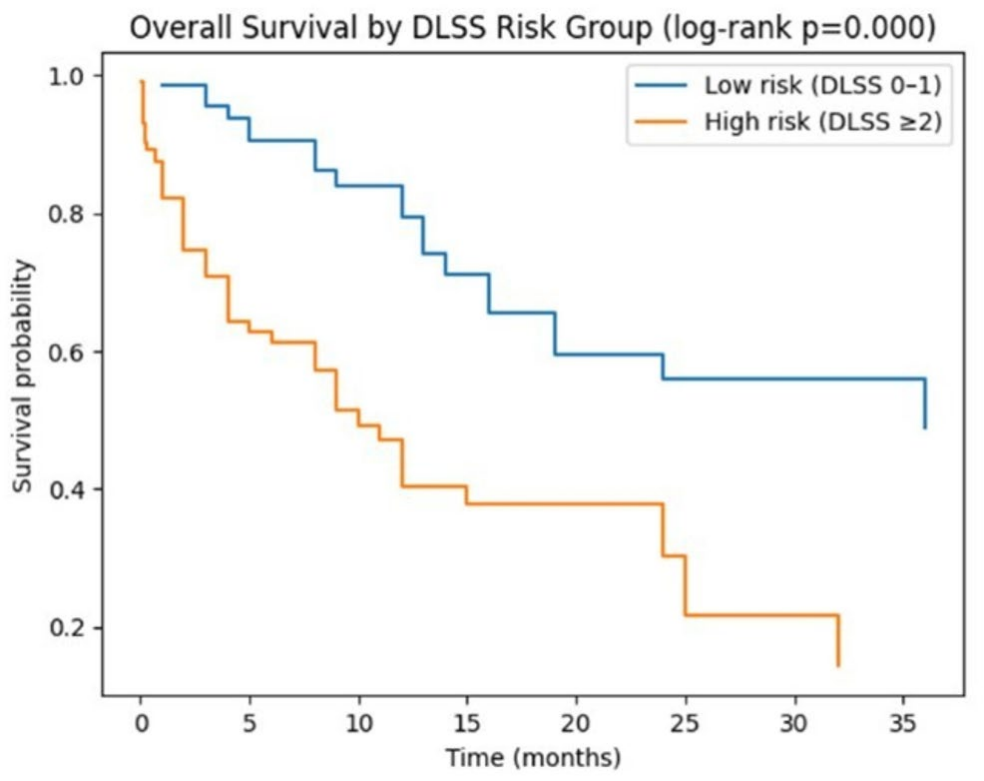
Kaplan–Meier survival stratified by DLSS risk category

At 12 months, mortality differed substantially across DLSS-defined risk categories. Patients with DLSS scores of 0–1 had a 12-month mortality of 23.4%, whereas those with DLSS ≥ 2 had a 12-month mortality of 72.3% (*p* < 0.001). Kaplan–Meier survival curves stratified by DLSS group demonstrated clear separation between risk categories (log-rank *p* < 0.001) (Fig. 5).

### Integrated interpretation

Taken together, these findings suggest that:

- Extent of resection showed the strongest association with survival within this surgically treated cohort.
- Lesion multiplicity demonstrated a smaller and context-dependent association with outcome after adjustment for other variables.
- The DLSS model provided internally derived perioperative risk stratification for early mortality following surgical treatment, although its clinical application requires cautious interpretation and external validation.

## Discussion

The present study provides long-term observational evidence from a surgically treated cohort of patients with brain metastases managed within a real-world multidisciplinary neuro-oncology environment. Within this two-decade institutional experience, intracranial lesion count alone did not emerge as the dominant variable associated with survival once operative extent and tumor biology were considered in multivariable analysis. Instead, survival patterns appeared to be more closely related to achievable cytoreduction and underlying biological disease behavior. These observations support an individualized treatment framework in which surgical considerations extend beyond simple lesion-count thresholds and incorporate broader clinical context [7–10].

Historically, the survival benefit of surgery was most clearly demonstrated in randomized trials evaluating patients with a single brain metastasis in the pre–stereotactic radiosurgery (SRS) era [11, 12]. Since that time, the therapeutic landscape has evolved substantially. Modern management strategies increasingly combine surgical resection, postoperative cavity-directed SRS, and systemic therapies with central nervous system activity. Consequently, contemporary guidelines emphasize multidisciplinary decision-making that incorporates neurological symptoms, mass effect, systemic disease status, and expected treatment integration rather than relying solely on numerical lesion thresholds [13–16]. The present findings are broadly consistent with this modern paradigm. Within the surgically selected cohort examined here, selected patients with multiple metastases experienced survival outcomes that did not differ dramatically from solitary-lesion cases once dominant-lesion resection and additional clinical variables were taken into account. However, this observation should be interpreted cautiously given the inherent treatment selection patterns that characterize retrospective surgical series.

A central observation of the present analysis is that extent of resection showed the strongest association with survival within the multivariable model. Patients undergoing gross total resection demonstrated lower mortality risk compared with those with subtotal resection. This finding aligns with longstanding neurosurgical principles emphasizing maximal safe cytoreduction as a cornerstone of local disease control, even in an era of advanced radiation delivery and increasingly effective systemic therapies [17–20]. Nevertheless, it is important to recognize that the relationship between extent of resection and outcome in retrospective cohorts may be influenced by surgical selection factors, tumor location, and overall disease burden. Consequently, the observed association should not be interpreted as definitive evidence of causal survival benefit but rather as a reflection of the complex interaction between tumor biology, surgical feasibility, and patient selection.

The association observed between lesion multiplicity and survival warrants careful interpretation. In the present model, multiple metastases demonstrated a modest statistical association with survival that appeared counterintuitive when considered in isolation. Similar paradoxical findings have occasionally been reported in surgically selected cohorts and likely reflect underlying selection patterns in which patients undergoing surgery for multiple lesions represent a biologically or clinically favorable subgroup—often characterized by dominant symptomatic lesions amenable to resection and otherwise controlled systemic disease. Accordingly, the direction of this association should be interpreted as context-dependent rather than biologically protective, and it underscores the limitations inherent to retrospective surgical datasets.

Beyond examining survival determinants, the present study explored the development of a pragmatic perioperative risk-stratification framework through the Dominant Lesion Surgery Score (DLSS). Derived from routinely available perioperative variables, DLSS demonstrated moderate discrimination for early mortality at both 6 and 12 months with stable internal performance after bootstrap validation. Calibration analyses suggested reasonable agreement between predicted and observed mortality probabilities, and decision-curve analysis indicated potential net benefit across clinically relevant threshold ranges. These findings suggest that DLSS may offer a structured method for exploring perioperative risk patterns within surgically treated patients, although the model should be regarded as exploratory and internally derived.

Existing prognostic systems for brain metastases—such as diagnosis-specific graded prognostic assessments—primarily incorporate demographic and systemic oncologic variables to estimate survival at a population level. While these models are valuable for general prognostic estimation, they are not specifically designed to address perioperative risk considerations within neurosurgical decision-making. In contrast, DLSS integrates surgical extent, lesion distribution, tumor subtype, and treatment context, thereby aligning prognostic estimation more closely with variables encountered during the perioperative management of brain metastases. Rather than replacing clinical judgment, such models may complement multidisciplinary discussion by providing an additional quantitative perspective in complex treatment scenarios.

Subtype-specific survival patterns observed in this cohort further emphasize the biological heterogeneity of metastatic disease. Patients with breast carcinoma metastases demonstrated comparatively more favorable outcomes, whereas gastrointestinal and other aggressive primary tumors tended to show shorter survival. These patterns are consistent with previously reported differences in systemic treatment responsiveness and intracranial disease behavior across tumor histologies [21–24]. Incorporating tumor biology into operative considerations therefore remains essential when evaluating potential surgical benefit.

Temporal analysis across the two treatment eras suggested that the principal evolution in modern practice has been strategic rather than purely survival-driven. Although only modest changes in median survival were observed between eras, the role of surgery appears increasingly integrated within multimodal treatment pathways. In contemporary practice, surgical intervention often facilitates neurological stabilization, relief of mass effect, histological diagnosis, and timely transition to radiosurgery or systemic therapy [13–16, 25]. This shift reflects a broader transformation in neurosurgical oncology, where the value of surgery is increasingly defined by its contribution to coordinated multidisciplinary care rather than by isolated survival outcomes.

From a clinical perspective, the present findings support consideration of dominant-lesion resection in carefully selected patients, including some individuals with multiple intracranial metastases when meaningful cytoreduction is achievable and integration with postoperative therapies is anticipated. Such an approach is consistent with contemporary guideline recommendations and reflects real-world multidisciplinary reasoning in modern neuro-oncology practice [7–9, 26–28]. In this context, DLSS may provide an exploratory framework for perioperative risk stratification that complements—rather than replaces—individualized clinical judgment.

Several limitations should be acknowledged. The retrospective single-center design introduces potential selection bias and limits external generalizability. Treatment heterogeneity across a prolonged study period reflects evolving real-world practice but may confound interpretation of temporal trends. Molecular and genomic tumor characteristics were not consistently available throughout the study period, preventing more detailed biological stratification. Additionally, postoperative treatments may introduce time-dependent effects that cannot be fully addressed in retrospective analyses. Finally, although DLSS demonstrated stable internal performance, external validation in independent cohorts is necessary before broader clinical implementation can be considered. Nonetheless, the extended follow-up, consistent institutional surgical approach, multivariable modeling, and combined assessment of discrimination, calibration, and decision-curve performance together provide a structured evaluation of perioperative risk patterns in surgically treated brain metastases.

## Conclusion

This long-term observational analysis from a surgically treated cohort suggests that intracranial lesion count alone may not adequately characterize postoperative risk among patients undergoing resection for brain metastases. Instead, outcomes appear to reflect a complex interaction between tumor biology, neurological presentation, achievable cytoreduction, and the broader multimodal treatment context. Within such a framework, resection of a dominant symptomatic lesion may remain a reasonable consideration in carefully selected patients, including some individuals with multiple metastases, when meaningful cytoreduction and integration with postoperative therapies are feasible.

The Dominant Lesion Surgery Score (DLSS) represents an exploratory perioperative risk-stratification framework derived from routinely available clinical variables. In this cohort, the model demonstrated moderate discriminatory performance for early postoperative mortality with acceptable internal calibration. These findings suggest that DLSS may provide a structured approach for exploring perioperative risk patterns following dominant-lesion resection. However, the score should be interpreted as an internally derived model rather than a definitive decision tool.

Overall, the present results support a shift away from rigid lesion-count–based exclusion criteria toward a more individualized and biology-informed perspective in the surgical management of brain metastases. External validation in independent and multicenter cohorts will be essential to determine the generalizability and potential clinical role of DLSS in contemporary neuro-oncology practice.

## Data Availability

The datasets generated and/or analyzed during the current study are available from the corresponding author on reasonable request.
